# Vitamin D and calcium-phosphorus in serum of children with celiac disease in a zone of high sunlight exposure

**DOI:** 10.3389/fped.2025.1594547

**Published:** 2025-08-05

**Authors:** Altinoy T. Kamilova, Nigora R. Alieva, Dilorom I. Akhmedova, Barno T. Abrorova

**Affiliations:** ^1^ Gastroenterology Department of Republican Specialized Scientific-Practical Medical Center of Pediatrics, Ministry of Health of Republic of Uzbekistan, Tashkent, Uzbekistan; ^2^ Hospital Pediatrics Department of Tashkent Pediatric Medical Institute, Tashkent, Uzbekistan

**Keywords:** celiac disease, children, vitamin d deficiency, parathormone, alkaline phosphatase, calcium, insolation

## Abstract

**Actuality:**

The relationship between vitamin D levels and celiac disease (CeD) in children remains controversial. Uzbekistan is a country where the average number of sunny days is more than 300 days. There are few studies on the vitamin D status of children with celiac disease in an area of high insolation.

**Aim of the study:**

To determine vitamin D status in children with CeD and to evaluate clinical and laboratory parameters in children depending on vitamin D levels.

**Patients and methods:**

We examined 60 children with first diagnosed CeD aged from 1 to 16 years, the average age was 6 ± 2.3 years. The diagnosis was established on the basis of ESPGHAN 2012 criteria. In all children serum 25(OH)D, calcium, phosphorus, parathormone, alkaline phosphatase was determined. The control group consisted of 31 children of identical age.

**Results:**

Children with CeD had significantly lower mean serum 25(OH)D levels (14.8 ± 1.04 ng/ml) compared to controls (45.1 ± 8.04 ng/ml; *p* < 0.001). Vitamin D deficiency (<20 ng/ml) was identified in 80% of patients with CeD, including 25% with levels <10 ng/ml. Vitamin D insufficiency was observed in 20%. Lower vitamin D levels were associated with more pronounced clinical features suggestive of metabolic imbalance, including stunting and growth retardation (observed in 41.7% and 43.8% of cases, respectively). Bone deformations were more frequent in vitamin children with D deficiency, with a significant inverse correlation between vitamin D levels and clinical bone manifestations. Serum alkaline phosphatase and parathormone levels were significantly higher in children with vitamin D deficiency and insufficiency (*p* < 0.05, *p* < 0.001), with inverse correlations between vitamin D and these markers.

**Conclusion:**

Children with celiac disease living in a region with increased sun light exposure showed a high prevalence of vitamin D deficiency. In our study, vitamin D deficiency in patients with celiac disease was associated with more severe clinical manifestations.

## 1 Introduction

Celiac disease (CeD) is a disorder caused by gluten intake in genetically predisposed individuals and characterized by atrophic enteropathy, which may present with a spectrum of both gastrointestinal and extraintestinal symptoms (1). It has been found to occur in approximately 1% of the population, and while it was previously identified more frequently in Europeans; studies in recent years have demonstrated a similar prevalence in Asians (2, 3). Our studies in 2021–2022 found that the incidence of CeD is 5.3% in at-risk groups (4).

Vitamin D is an essential micronutrient involved in the regulation of calcium homeostasis and bone metabolism. Deficiency of vitamin D during early childhood has been implicated in the development of various autoimmune disorders, including celiac disease. Vitamin D may also be a potential protective factor for CeD due to its role in the regulation of the immune system (5, 6). Vitamin D status plays an important role in the pathogenesis of intestinal diseases characterized by malabsorption and maldigestion syndromes, particularly because vitamin D is primarily absorbed in the duodenum and jejunum in the presence of bile acids (7–9).

There are conflicting data in the literature on the incidence of vitamin D deficiency in children with CeD. However, most studies have reported low vitamin D values in Turkey, the Russian Federation, and Iran (10, 11). Although vitamin D is often referred to as the “sunshine vitamin” a deficiency has been documented in many Asian countries, including China and India (12). As demonstrated in our previous studies, despite the fact that Uzbekistan has more than 300 sunny days per year, normal serum vitamin D levels were observed in only 18% of children even during the summer months (13, 14).

Taking into account that in Uzbekistan at the same time CeD is characterized by the predominance of severe forms with intestinal manifestations (4), the aim of the study was to determine the status of vitamin D in children with newly diagnosed celiac disease and to evaluate the impact of the degree of vitamin D deficiency on the clinical and laboratory manifestations of the disease.

## 2 Methods

### 2.1 Study design

This is cross-sectional study investigating status vimain D in children new diagnosed CeD, who were refered to the Gastroenterology department of the Republican Specialized Scientific and Practical Medical Center of Pediatrics of the Ministry of Health of the Republic of Uzbekistan during the period from Septeber 2016 till Desember 2017 year. None of the children had a history of vitamin D supplementation or any comorbid conditions that could have contributed to vitamin D deficiency or insufficiency (such as bone deformations or parathyroid gland diseases).

Children presenting with gastrointestinal symptoms indicative of celiac disease, who were referred to the Department of Pediatric Gastroenterology from community clinics or peripheral hospitals, were assessed based on the 2012 diagnostic guidelines of the European Society for Pediatric Gastroenterology, Hepatology, and Nutrition (ESPGHAN) (15). The study excluded participants who: (i) had a confirmed diagnosis of CeD prior to referral; (ii) were following a gluten-free diet at the time of enrollment; or (iii) did not provide informed consent to participate.

The study was carried out in accordance with international ethical standards and received approval from the Ethics Committee of the Republican Specialized Scientific-Practical Medical Center of Pediatrics (RSSPMCP) (approval number IP-2016, dated May 17, 2016). Written informed consent was obtained from the legal guardians of all participants, and the study complied with the principles of the Declaration of Helsinki. The physical development of children was assessed using reference tables of anthropometric indicators proposed by experts from the World Health Organization, using the WHO Anthro, WHO AnthroPlus programs (16).

### 2.3 Diagnostic work-up

For symptomatic children, the no-biopsy diagnostic pathway—applicable when anti-tissue transglutaminase IgA (anti-tTG IgA) levels exceeded 10 times the upper limit of normal and endomysial antibodies (EMA IgA) were positive in a second serum sample—was considered (11). However, due to the unavailability of EMA IgA testing in our country, all children with positive anti-tTG IgA results were advised to undergo esophagogastroduodenoscopy (EGDS) with histological examination of the duodenal mucosa.

Consequently, CD diagnosis was based on positive serologic findings (anti-tTG IgA) in combination with Marsh grade 2 or higher histopathological changes. In IgA-deficient individuals, anti-tTG IgG was measured, and a diagnosis was confirmed if histologic findings were consistent with CD. All patients diagnosed through this diagnostic process were also genotyped for HLA-DQ2 and DQ8.

Total serum IgA was quantified using a two-step sandwich ELISA with monoclonal anti-IgA antibodies (Cat. No. A-8666, Vector-BEST, Novosibirsk, Russia). In cases of reduced IgA levels, total IgG and anti-tTG IgG were also measured. Total IgG was assessed with a similar sandwich ELISA kit using monoclonal antibodies (Cat. No. A-8662, Vector-BEST, Novosibirsk, Russia). Quantitative determination of anti-tTG IgA (or anti-tTG IgG, when applicable) was performed using ELISA kits from Orgentec Diagnostika GmbH (Cat. No. 416-5400A, ORG 540G, Mainz, Germany).

HLA genotyping was conducted using sequence-specific primer polymerase chain reaction (SSP-PCR) with DQ kits targeting DQA1*05, DQB1*02, DQA1*0301, DQB1*0302, DQA1*0505, and DQB1*0202 alleles to detect DQ2.5, DQ2.2, and DQ8 haplotypes (Celiacstrip HLA DQ2DQ8, OPERON, Inmuno and Molecular Diagnostics, Caparoca, Spain).

25(OH)D and parathyroid hormone (PTH) in the serum were determined by the ELISA method on the Elecsys apparatus (Switzerland). Total and ionized calcium, inorganic phosphorus and alkaline phosphatase (ALP) were determined biochemically. The results were assessed in accordance with the recommendations of the International Society of Endocrinologists (2011): vitamin D deficiency—less than 20 ng/ml (less than 50 nmol/L); vitamin D insufficiency—21–29 ng/ml (51–75 nmol/L); normal vitamin D content—30–100 ng/ml (76–250 nmol/L). A concentration below 10 ng/ml (less than 25 nmol/L) is interpreted as severe vitamin D deficiency. A level above 100 ng/ml (more than 250 nmol/L) is considered as excess vitamin D (17). The control group consisted of 31 children aged 3–15 years (mean age: 7 ± 2.1 years) who had not received vitamin D supplementation. The group included 14 boys and 17 girls.

Histological evaluation of duodenal mucosal changes was conducted by experienced histopathologists using the Marsh–Oberhuber classification system. According to this grading, Marsh–Oberhuber grade 2 is characterized by an increased number of intraepithelial lymphocytes along with crypt hyperplasia. Marsh–Oberhuber grade 3 (subtypes a, b, and c) indicates partial, subtotal, or total villous atrophy, respectively, in combination with intraepithelial lymphocyte infiltration and crypt hyperplasia.

### 2.4 Statistical analysis

Statistical analysis was carried out using Microsoft Excel with built-in statistical tools and STATISTICA 10.0 software [StatSoft, Inc. (2011)]. Methods of descriptive and inferential statistics were employed. This included calculation of relative frequencies (percentage, %), measures of central tendency and dispersion (arithmetic mean [M], standard deviation [*σ*], and standard error [m), and comparison of means using Student's t-test (t). A *p*-value of ≤ 0.05 was considered statistically significant. Correlations between variables were analyzed using Pearson's correlation coefficient. Descriptive statistics are presented as absolute numbers and percentages (%).

The sample size was not calculated preliminarily, a continuous study of children who came to our center with the first established CeD was carried out. Studies were carried out with the written consent of their parents.

Missing data were handled using the Complete Case Analysis method, in which rows/columns containing gaps were excluded from the data set. Statistical analysis was performed using GraphPad Prism (version 9.3.1, 2021). Descriptive statistics included the calculation of the mean (M), standard deviation (SD), median (Me), and interquartile range (Q1; Q3). The Kolmogorov–Smirnov test was used to evaluate the normality of data distribution. Group comparisons were conducted using either Student's t-test for normally distributed variables or the Mann–Whitney *U*-test for non-normally distributed data.

For measuring the strength and direction of the relationship between two variables Pearson's correlation coefficient was used. Categorical variables were expressed as absolute and relative values. 95% CI for the proportion was calculated using the Wald normal approximation method. The differences were considered statistically significant at *p* < 0.05, the calculation was made by the two-sided *p*-value.

## 3 Results

### 3.1 Study population: clinical features in children with celiac disease depending on vitamin D status

During the study period, celiac disease was diagnosed in 69 children who came to our centre, of whom 9 refused the study, therefore, the study population consisted of 60 children (age range: 1–16 years, 60 children with celiac disease aged from 1 year to 16 years were examined, the average age was 6 ± 2.3 y including 36 boys and 24 girls. According to the distribution of the decrease in vitamin D: deficiency was detected in 48 (80%) patients with CeD, and in every fourth patient (15/25%) extremely low levels were established (below 10 ng/ml). Insufficient vitamin D content was detected in 12 patients with CeD (20%) (*p* < 0.05) (Table 1).

**Table 1:** Vitamin D levels in the examined children.

Groups	Vitamin D3, ng/ml
CeD (*n* = 60)	14.8 ± 1.04***
Control (*n* = 31)	45.1 ± 8.04

Reliability of data for the control group: *Р < 0.05; **P < 0.01; ***P < 0.001.

Comparison of clinical manifestations in children with CeD depending on the deficiency and insufficiency of vitamin D demonstrated the severity of bone deformations and metabolic disorders in group with vitamin D deficiency compared to insufficiency: bone pain, dull hair, dry skin, hair loss (Table 2).

**Table 2:** Clinical features in children with celiac disease depending on vitamin D status (*n* = 60).

Symptoms	Vitamin D deficiency (*n* = 48)		Vitamin D insufficiency (*n* = 12)	
	Abs.	%	Аbs.	%
PEI:				
Mild	6	12.5 ± 4.8	2	16.7 ± 2.3
Moderate	16	33.3 ± 6.8	1	8.3 ± 7.8*
Severe	5	10.4 ± 4.4	0	0.0 ± 0.0*
Bone pain	40	83.3 ± 5.4	4	33.3 ± 13.6*
Dental caries	30	62.5 ± 6.9	6	50.0 ± 14.4
Dental deformity	47	97.9 ± 2.1	5	41.7 ± 14.2*
Dull hair	46	95.8 ± 2.9	7	58.3 ± 14.2*
Hair loss	45	93.8 ± 3.5	5	41.7 ± 14.2*
Muscle hypotonia	46	95.8 ± 2.9	5	41,7 ± 14,2*
Dry skin	45	93.8 ± 3.9	5	41.7 ± 14.2*
Lethargy	44	91.7 ± 3.5	8	66.7 ± 13.6
Weakness	43	89.6 ± 4.4	9	75.0 ± 12.5
Adynamia	17	35.4 ± 6.9	3	25.0 ± 12.5
Sweating	48	100.0 ± 0.0	12	100.0 ± 0.0
Tachycardia	31	64.6 ± 6.9	5	41.7 ± 14.2

* Reliability of data between indicators for vitamin D deficiency and insufficiency (*P* < 0.05).

### 3.2 Nutritional status children with CeD depending vitaaamin D levels

When studying anthropometric data depending on vitamin D deficiency and insufficiency, we found that with its deficiency, every third patient with CeD suffered from severe weight deficiency, growth retardation of more than 3 SD was established in 41.7 ± 7.1% of cases. Whereas with insufficiency, these indicators were 8.3 ± 10.8% and 16.7 ± 10.8%, respectively (Table 3).

**Table 3:** Length/height and weight indicators in children with bowel diseases depending on vitamin D levels.

Vitamin D level	Short stature and growth retardation, SD				Underweight and low body weight, SD			
	−2SD-3SD		Lower −3SD		−2SD–−3SD		Lower −3SD	
	abs.	%	abs.	%	abs.	%	abs.	%
Deficiency								
CeD, *n* = 48	21	43.8 ± 7.2	20	41.7 ± 7.1	16	33.3 ± 6.8	14	29.2 ± 6.7
Insufficiency								
CeD, *n* = 12	3	25.0 ± 12.5	2	16.7 ± 10.8	2	16.7 ± 10.8	1	8.3 ± 7.9

### 3.3 Results of studies of calcium, phosphorus, parathyroid hormone (PTH) and alkaline phosphatase (ALP) in the blood serum of children with celiac disease

The results of studies of calcium-phosphorus metabolism and its regulators demonstrated a relationship between the level of vitamin D deficiency and a deficiency of total and ionized calcium, phosphorus in patients with CeD, as well as an increase in alkaline phosphatase and parathyroid hormone in patients with CeD. An increased level of parathyroid hormone (PTH) was observed among patients with CeD, exceeding the values of the control group by 3.2 times (*p* < 0.001). Thus, the alkaline phosphatase (ALP) indicators were increased by 2.3 times with vitamin D deficiency and by 1.2 times with insufficiency; parathyroid hormone—by 3.2 times with vitamin D deficiency and by 2.6 times with vitamin D insufficiency (*p* < 0.05, *p* < 0.001) (Table 4).

**Table 4:** Biochemical parameters in children with celiac disease depending on vitamin D levels.

Vitamin D level	Са	Ionized Ca	ALP	Phosphorus	PTH
Control group *n* = 31	2.35 ± 0.08	1.2 ± 0.04	95.5 ± 1.4	1.48 ± 0.05	9.1 ± 0.5
Deficiency, *n* = 92					
CeD, *n* = 48	2 ± 0.04**	0.96 ± 0.07**	219.5 ± 2.9***	0.98 ± 0.05***	29.3 ± 1.9***
Insufficiency, *n* = 65					
CeD, *n* = 12	2.2 ± 0.03^^	1.1 ± 0.02*^	179.1 ± 2.9**^^	1.13 ± 0.02***^	24.6 ± 3.2***^

Reliability of indicators to the norm (*P < 0.05; **P < 0.01; ***P < 0.001). Reliability of data between indicators for vitamin D deficiency and insufficiency (^*P* < 0.05; ^^*P* < 0.01).

A reliable significant difference in the levels of total, ionized calcium and phosphorus was also established in vitamin D deficiency compared to its insufficiency. Analysis of the correlation relationship between vitamin D concentration and biochemical parameters characterizing phosphorus-calcium metabolism and the level of parathyroid hormone showed that in CeD, an inverse correlation was found between vitamin D content and the level of alkaline phosphatase (respectively r = −0.710 and r = −0.623, r = −0.589) and parathyroid hormone (respectively r = −0.610 and r = −0.659, r = −0.623) (Table 5).

**Table 5:** Correlation indicators of the blood concentration of vitamin D3 with the concentration of parathyroid hormone, calcium, phosphorus and alkaline phosphatase.

Pathology	PTH	Calcium	Phosphorus	ALP
Celiac disease	−0.610	0.106	−0.365	−0.710

## 4 Discussion

Our studies have demonstrated a profound deficiency of vitamin D in children with CeD in the active phase of the disease (14.8 ± 1.04 ng/ml), in parallel with increased levels of parathyroid hormone and alkaline phosphatase, and reduced concentrations of calcium and phosphorus in the blood serum. The results of our studies were similar to those of Turkish researchers (10), who reported that the average serum 25(OH)D level in children and adolescents with CeD was 18.5 ng/ml, based on a large sample of patients (*n* = 6,717).

The results of a meta-analysis also confirmed that vitamin D levels in pediatric patients with CeD were lower than in healthy individuals (18). It was found that patients with CeD from South Asia had a significantly higher prevalence of vitamin D deficiency compared to Caucasian patients (70.8% vs. 32.8%, *p* = 0.002) (19).

According to van der Mei et al. (20), in the absence of vitamin D supplementation, vitamin D status is largely determined by endogenous synthesis, which is affected by skin pigmentation—a relevant factor for children in us.

As we have previously noted, the determination of vitamin D levels in healthy children in our Republic during the summer season demonstrated a high percentage of insufficiency and deficiency (82%) (13). Our data are close to the findings of Hataikarn Nimitphong and Michael F. Holick (21), who reported that vitamin D deficiency reached about 70% in South Asia and varied from 6% to 70% in Southeast Asia.

Most of Brazil's territory lies in the tropical zone, and only its southernmost part lies in the subtropical zone. This geographic location results in high solar radiation. A study of 599 children and adolescents aged 6–19 years found that 62 (10.4%) had vitamin D deficiency, 257 (42.9%) had insufficiency, and 280 (46.7%) had sufficient levels. Thus, suboptimal serum vitamin D levels (<30 ng/ml) were found in 53.3% (*n* = 319) of participants (22).

For many years, it was assumed that living in regions with abundant sunlight guaranteed sufficient vitamin D levels. However, accumulating evidence indicates that vitamin D insufficiency remains a commonly underestimated health concern, even in sunny countries.

Previous studies have shown that the level of vitamin D in the group of patients with CeD negatively correlates with the severity of symptoms, that is, the lower the level of vitamin D, the more severe the symptoms in patients with CeD (23). Our own findings align with this, demonstrating that children with vitamin D deficiency tend to have lower weight and height, along with more frequent metabolic and bone manifestations.

In previous retrospective studies conducted with a limited number of cases, the incidence of vitamin D deficiency in patients with CeD ranged from 27% to 70% (24, 25, 26). Several studies have reported conflicting results regarding the serum 25(OH)D levels at the time of CeD diagnosis (27, 28, 29). Ahlawat et al. (29) reported that there was no difference between 25(OH)D levels in patients newly diagnosed with CeD and controls. Similarly, Villanueva et al. (30) reported that vitamin D levels in patients with CeD were not different from controls. Lerner et al. (31) compared vitamin D levels in patients newly diagnosed with CeD and found no differences. In contrast, in the study by Lionetti et al. (32), vitamin D levels in children and adolescents with CeD were lower at the time of diagnosis compared to controls. Vitamin D deficiency is thought to be associated with decreased expression of the vitamin D receptor and epithelial barrier proteins E-cadherin and claudin-2, which play an important role in children with CeD in correlation with histological indicators of disease severity (33).

Malaguarnera's findings further supported the role of vitamin D in intestinal homeostasis, indicating that on the role of vitamin D in maintaining intestinal homeostasis through local synthesis of 1α,25(OH)2D3 and expression of Vitamin D Receptor, emphasizing the critical importance of optimal 1α,25(OH)₂D₃ levels, as this active form of vitamin D is involved in a range of regulatory processes, including not only calcium absorption, but also immune defense, preservation of epithelial barrier integrity, and modulation of the intestinal microbiota. This role is receiving increased attention, since an unbalanced microbiota may be associated with a number of negative health disorders, such as inflammation, allergic reactions, autoimmune diseases, heart disease, obesity and metabolic syndrome (34). The potential positive role of vitamin D on dendritic cells has been recently highlighted, demonstrating a close relationship between suboptimal vitamin D levels and the occurrence and progression of many autoimmune diseases (35).

As we mentioned, the time of year has a certain significance for vitamin D indicators, since when determining the values of vitamin D in the blood serum of conditionally healthy children, higher values were found in the summer (36). It is important to acknowledge the limitations of our study, particularly the relatively small sample size, which was primarily due to financial constraints. The study was conducted over the course of one year, and we did not analyze seasonal changes in serum vitamin D levels in children with CeD, due to the lack of statistically significant differences across seasons. Our previous studies showed that in summer, normal vitamin D levels were observed in only 18% of healthy children, while 17% had vitamin D deficiency and 65% had insufficiency (13). Also one limitation of our study is the use of data collected between 2016 and 2017. Although these data were obtained several years ago, they still provide relevant insights into the clinical and nutritional status of children with celiac disease prior to the implementation of updated diagnostic and therapeutic standards. The temporal gap is acknowledged and was considered in the interpretation of results.

## 5 Conclusions

A high proportion of children with celiac disease in a region with increased insolation were found to have vitamin D deficiency, along with altered biochemical parameters, including increased parathyroid hormone and alkaline phosphatase levels and decreased total and ionized calcium and phosphorus. In our study, vitamin D deficiency in children with celiac disease was associated with markers of increased disease severity, including metabolic bone abnormalities and lower physical development scores.

Considering the association observed between lower vitamin D levels and the severity of clinical symptoms in children with celiac disease, it may be advisable to assess vitamin D status in children residing in regions with high sunlight exposure and to consider differentiated correction strategies based on individual needs.

## Data Availability

The original contributions presented in the study are included in the article/Supplementary Material, further inquiries can be directed to the corresponding author.
